# Cholesteric Chiral Molecular Tweezer for Rapid Detection of F^−^ in Food Samples

**DOI:** 10.3390/molecules27031098

**Published:** 2022-02-07

**Authors:** Zhe Liu, Hong Wang, Zhu Chen, Ying Ye, Yuwei Wang

**Affiliations:** 1 College of Agriculture and Animal Husbandry, Qinghai University, Xining 810016, China; lz239880356@163.com (Z.L.); chenzhu202103@163.com (Z.C.); 2 College of Chemical Engineering, Qinghai University, Xining 810016, China; whong714@126.com; 3 State Key Laboratory of Plateau Ecology and Agriculture, Qinghai University, Xining 810016, China

**Keywords:** cholesteric molecular tweezer, fluoride ions, naked-eye detection, specific recognition, food samples

## Abstract

Chiral cholesteric molecular tweezer 7d was synthesized and its influences on changes in the ultraviolet (UV) and fluorescence spectra of various anions were investigated. The results displayed that molecular tweezer 7d selectively recognized F^−^ ions in dimethyl sulfoxide with a detection limit of 5.14 μmol/L, while other anions had little interference. On this basis, a method for the rapid detection of F^−^ ions by host molecular tweezer 7d was established, and the naked-eye detection of F^−^ was realized through the unique yellow color of the complex solution. According to the determination of F^−^ ions in real food samples, it was proved that the established method had good application prospects in F^−^ ion detection.

## 1. Introduction

It is well known that anions are abundant in our daily life and play an irreplaceable role, especially in biological processes, environmental detection, and clinical medicine fields [1,2,3]. To date, researchers have designed and synthesized a series of anionic receptor compounds and applied them to the selective recognition and detection of specific anions. Among various anions examined, fluorine ion (F^−^) has attracted increasing attention due to its special role in life bodies. Fluorine is an indispensable trace element for human growth and development [4], and plays a pivotal role in the prevention of dental caries and the treatment of osteoporosis. Therefore, trace fluoride is widely used as an additive in toothpaste and water to achieve the purpose of strengthening bones and protecting teeth. However, fluoride is also a cumulative toxic substance. Once excessive F^−^ ions are ingested by the human body, they may induce cell apoptosis, decrease the immune function of the body [5], and even cause damage to the bones, heart, kidneys, and other tissues and organs [6,7], thus greatly harming human health. According to the regulations of the World Health Organization, the limit of F^−^ ion content in daily drinking water is 1.5 mg/L [8]. In China’s current drinking water sanitation standard, F^−^ ion concentration should not exceed 1.0 mg/L to prevent F^−^ ion poisoning [9]. Hence, efficient F^−^ ion detection is of great significance to human health and chemical environment.

In recent years, many traditional detection methods have been developed for F^−^ ion identification, including gas chromatography [10], the ion selective electrode method [11], ^19^F^−^ nuclear magnetic resonance analysis [12], and high performance liquid chromatography (HPLC) method etc. [13]. However, these methods greatly rely on large-scale precision instruments and professional operation technical personnel, as well as complicated pretreatment of samples, thus greatly restricting their practicability. Therefore, it is important to develop new methods for F^−^ ion detection with high sensitivity and selectivity. At present, a large number of studies have reported that the photochemical response recognition technique can promote rapid and efficient selectivity between the guest and receptor, promoting the realization of qualitative and quantitative detection of specific ions [14]. Generally, in the anionic recognition system, amides [15,16], thioureas [17,18], pyridines [19,20], and indoles [21,22] are considered desirable ligands with high sensitivity and selectivity. In addition, cholesteric compounds are of great interest to researchers due to their simple preparation method and mild reaction process, as well as flexible and multifunctional recognition sites in their structures. Moreover, different types of anion recognition receptors can be designed through the modification of hydroxyl and carboxyl groups in the steroidal skeleton. As a result, cholesteric compounds have attracted considerable global attention [23]. Liu et al. [24] designed and synthesized two new tweezer-like anionic artificial receptors using deoxycholic acid as the spacing group under microwave radiation for the first time. They found that these compounds had a recognition ability for guest anions F^−^, Cl^−^, Br^−^, I^−^, Ac^−^, and H_2_PO_4_^−^, and that the host and guest could form a 1:1 type supramolecular complex by multiple hydrogen bonds. Chahar et al. [25] performed closed-loop modification on cholesteric diimidazole derivatives and synthesized a new deoxycholic-acid ring-like receptor; when naphthalene was used as the spacer, the receptor recognized F^−^ ions with high sensitivity and selectivity.

In this study, on the basis of our previous work [26], we synthesized a target cholesteric chiral molecular tweezer artificial receptor 7d using the microwave radiation technique through a series of chemical reactions with deoxycholic acid as the raw material. The recognition performance of the receptors toward anions was determined by ultraviolet and fluorescence spectrum analysis, and the probable recognition mechanism was speculated via nuclear magnetic titration and molecular simulation. Furthermore, a rapid and quantitative determination method for F^−^ ions by chiral molecular tweezer was established and applied to F^−^ detection in food samples. It is expected that the presented cholesteric compounds can provide a new method for the rapid determination of F^−^ ions.

## 2. Results

### 2.1. Selective Recognition of Anions by Host Molecular Tweezer 7d

**Figure 1:**
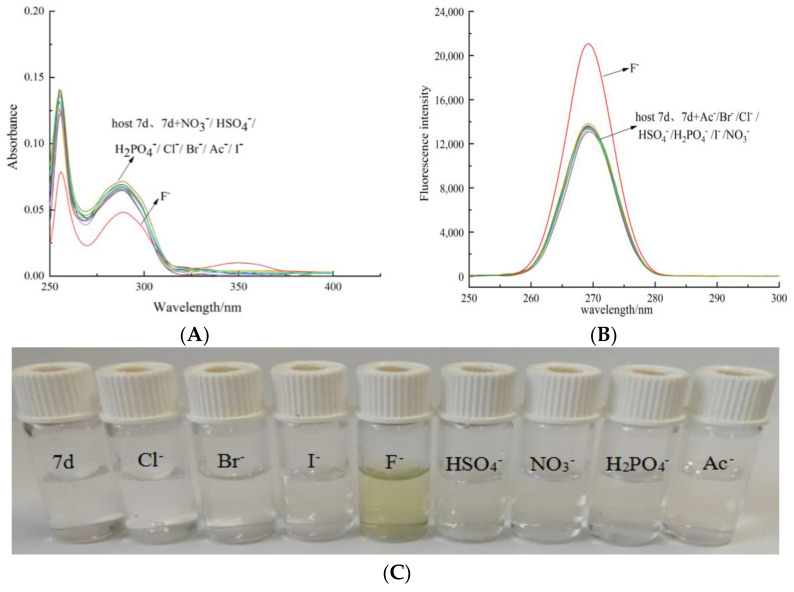
(**A**) UV–vis absorption spectra, (**B**) fluorescence emission spectra, and (**C**) color changes of chiral molecular tweezer 7d (20 μmol/L) in the presence of different anions (1 × 10^−3^ mol/L).

As shown in Figure 1A, when different anions are added to host molecular tweezer 7d solution, only F^−^ ions significantly change UV–vis absorption spectrum. The intensities of the absorption peaks at 255 and 288 nm decrease significantly, and a new absorption peak appears at 350 nm. In contrast, the addition of other anions does not cause significant changes in UV–vis absorption spectrum of host molecular tweezer 7d solution. It should be noted that the solution changes from colorless to distinctly yellow after the interaction with F^−^ ions, while other anions show no color change (Figure 1C). This may be due to the ability of chiral molecular tweezer 7d to complex with F^−^ ions, which promotes a color change. Therefore, host molecular tweezer 7d can achieve naked-eye specific recognition of F^−^ ions. In order to further investigate the selective recognition effect of chiral molecular tweezer 7d towards anions, the emission properties of the above anions were investigated by fluorescence spectroscopy under the same conditions. As shown in Figure 1B, after the addition of various anions, only F^−^ ions provide the highest fluorescence intensity value at 273 nm. This may be attributed to the enhanced rigidity of the complex formed by chiral molecular tweezer 7d and F^−^ ions relative to that of the molecular tweezer 7d solution, thus enhancing the fluorescence intensity [27]. Hence, host 7d and F^−^ ions produce good selectivity, which can achieve highly selective recognition.

### 2.2. Influences of Coexisting Ions and Response Time Test

**Figure 2:**
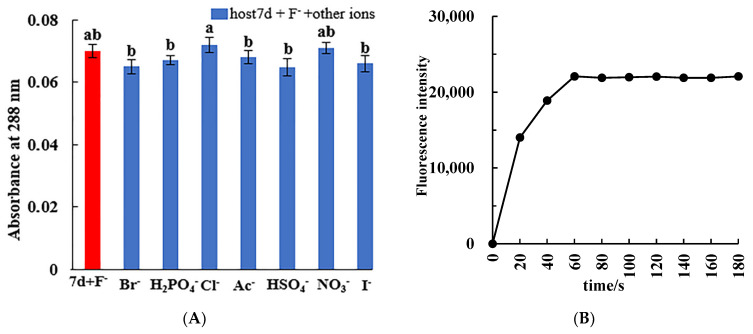
(**A**) Histogram of absorbance values at 288 nm of chiral molecular tweezer 7d solution (20 μmol/L) after the addition of F^−^ ion and other anions (8.33 × 10^−4^ mol/L); (**B**) Fluorescence intensity values at 273 nm of molecular tweezer 7d solution (20 μmol/L) after the addition of F^−^ (1 × 10^−3^ mol/L).

In order to further evaluate the selectivity of molecular tweezer 7d to F^−^ ions in complicated environment, the anti-interference ability of molecular tweezer 7d in dimethyl sulfoxide (DMSO) solution in the presence of other anions was studied. As shown in Figure 2A, addition of other competitive anions to the complex system shows little interference to UV–vis absorbance value of the characteristic absorption peak at 288 nm compared to that of the original complex system without other anions, and the relative error is less than 10% (Table 1). The obtained results indicated that other coexisting interfering anions had little effect on the recognition process of F^−^ ions by the host molecular tweezer 7d. Thus, the host molecular tweezer 7d shows good anti-interference performance in the selective recognition of F^−^ ions, and has wide environmental adaptability and practicability.

In order to determine the response time of compound 7d to recognize F^−^ ions, the fluorescence intensity at 273 nm was measured after adding molecular tweezer 7d into F^−^ ionic solution (Figure 2B). As can be seen from the graph, the fluorescence intensity of cholesteric molecular tweezer 7d increased rapidly with the addition of F^−^, and finally tended to be stable after 60 s. Therefore, this method can be applied to fast identification and detection of F^−^.

### 2.3. UV Titration Test at Different Concentrations of F^−^ Ions by Host Molecular Tweezer 7d

**Figure 3:**
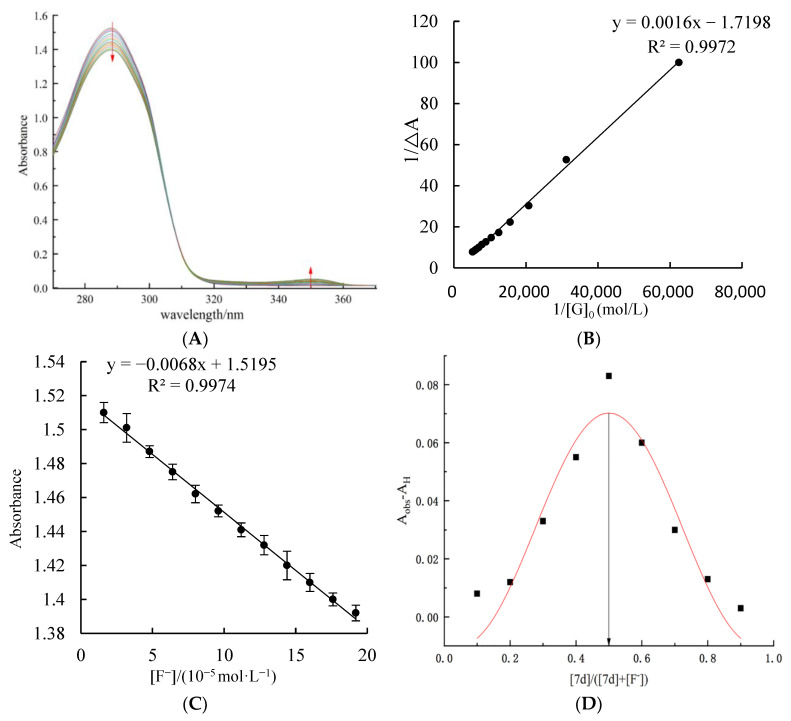
(**A**) UV–vis spectrum titration for chiral molecular tweezer 7d (5.0 × 10^−4^ mol/L) and different concentrations of F^−^ ions (0–192 μmol/L); (**B**) Benesi–Hildebrand linear fitting of chiral molecular tweezer 7d (5.0 × 10^−4^ mol/L) and different concentrations of F^−^ ions (0–192 μmol/L); (**C**) Standard curve of the absorbance value versus the concentration of F^−^ ions (16–192 μmol/L) after drop-wise addition F^−^ ions into the chiral molecular tweezer 7d solution (5.0 × 10^−4^ mol/L); (**D**) Job’s plot of the interaction system between chiral molecular tweezer 7d and F^−^ ions.

In order to further understand and verify the interaction between host molecular tweezer 7d and F^−^ ions, a UV titration test for different concentrations of F^−^ ions was carried out. As shown in Figure 3A, in the absence of F^−^ ions, a characteristic absorption peak at 288 nm for host molecular tweezer 7d solution is observed. After the addition of F^−^ ions, the interaction between the host molecular tweezer 7d and F^−^ ions causes a weakening trend of the intensity of the characteristic absorption peak at 288 nm; meanwhile, a new absorption peak appears at 350 nm, and its intensity gradually increases with increasing F^−^ ion concentration. Therefore, the host–guest complex is formed in the titration process. Within an F^−^ ion concentration range of 1.60 × 10^−5^ to 1.92 × 10^−4^ mol/L, according to the Hildebrand–Benes equation [28], the graph of 1/[G_0_] versus 1/ΔA was plotted. Figure 3B shows that through fitting, a good straight-line relationship is obtained (R^2^ = 0.997). Hence, the host molecular tweezer 7d and F^−^ ions generate a 1:1 type complex. According to the intercept 1/a and slope 1/a•Ka of the straight line, the association constant Ka of the complex was calculated as 1719 L/mol. In addition, by plotting the graph of UV–vis absorbance value versus F^−^ ion concentration during the complex formation process (Figure 3C), the absorbance value of the chiral molecular tweezer 7d has a good linear relationship with F^−^ ion concentration in the range of 16–192 μmol/L. Through fitting, the standard curve at 288 nm is y = −0.006x + 1.519. According to the formula, LOD = 3 σ/k, where σ is the standard deviation and k is the slope of the standard curve, the detection limit of F^−^ ions by the host molecular tweezer 7d is 5.14 μmol/L, which is far lower than the standard of F^−^ ions in drinking water formulated by the US Environmental Protection Agency (EPA) (210 μM) [29]. Compared with reported detection sensors of F^−^ ions (Table 2), the host molecular tweezer 7d has a higher recognition sensitivity to F^−^ ions, and the detection system is intuitive and fast, requiring no complex processes or expensive instruments. Therefore, the chiral molecular tweezer 7d can be used for the rapid detection of F^−^ ions within this concentration range.

The continuous variation method with a constant molar ratio (Job test) is another effective method for determining the coordination ratio of host molecular tweezer 7d to F^−^ ions [30]. As shown in Figure 3D, when the molar ratio of chiral molecular tweezer 7d to 7d + F^−^ ions is 0.5, the complex of 7d + F^−^ ions has the highest absorbance value. Hence, the chiral molecular tweezer 7d complexes with F^−^ ions following a molar ratio of 1:1.

**Table 2:** Comparison of various recognition sensors for F^−^.

Compound	Solvent	Detection Limit	Ref.
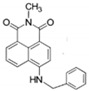	CH_3_CN	20 × 10^−6^ M	[31]
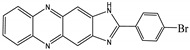	DMSO	6.2 × 10^−6^ M	[32]
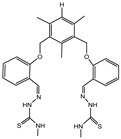	DMSO	2 × 10^−5^ M	[33]
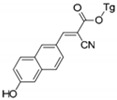	PBS buffer	8.54 × 10^−6^ M	[34]
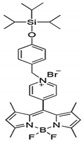	PBS buffer	2 × 10^−5^ M	[35]
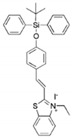	PBS buffer	8 × 10^−5^ M	[36]
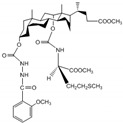	DMSO	5.14 × 10^−6^ M	This work

### 2.4. Microstructure Observation and Energy Spectrum Imaging Analysis of the Complex

**Figure 4:**
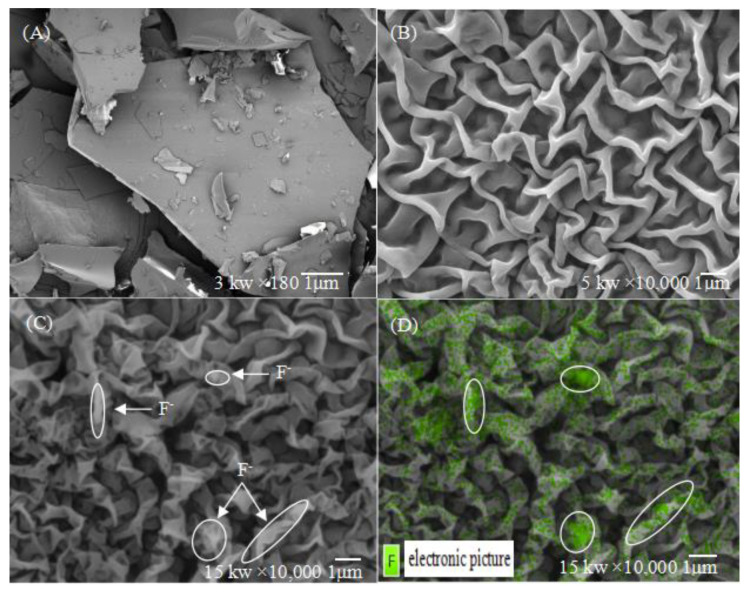
(**A**) FESEM image (×180, 3 kw) of the host compound 7d without the addition of F^−^ ions; (**B**) FESEM image (×10,000, 5 kw) of the host compound 7d in the presence of F^−^ ions; (**C**) FESEM image (×10,000, 15 kw) of the host compound 7d in the presence of F^−^ ions; (**D**) Energy spectrum image (×10,000, 15 kw) of the host compound 7d in the presence of F^−^ ions.

Figure 4 shows field emission scanning electron microscope (FESEM) images of the complex formed by host molecular tweezer 7d and F^−^ ions. In the absence of F^−^ ions (Figure 4A), the host molecular tweezer 7d mainly accumulates producing a form of dense blocks. After complexing with F^−^ ions (Figure 4B), the structure of the host molecular tweezer 7d changes significantly, and a regular wrinkled network structure is formed. The uplift regions of the structure show the morphology of banded cavities, which encapsulate F^−^ ions and forms stable molecular aggregates [37]. In order to further verify that the encapsulated particles are F^−^ ions, SEM imaging (Figure 4C) and corresponding energy spectrum imaging (Figure 4D) of the sample were obtained using a JSM 7900F high resolution transmission scanning electron microscope (HRTEM) at an acceleration voltage of 15.0 kV. As shown in Figure 4C, when the host molecular tweezer 7d reacts with F^−^ ions, a large number of F^−^ ions enter into the host’s cavities, generating many highlighted bands in the image. Based on the spectral imaging analysis, the green regions in Figure 4D represent the distribution of F^−^ ions. Additionally, the vast majority of F^−^ ions have been embedded into the cracked cavities successfully and wrapped by the host compound 7d, forming a stable complex compound.

### 2.5. Recognition Mechanism

**Figure 5:**
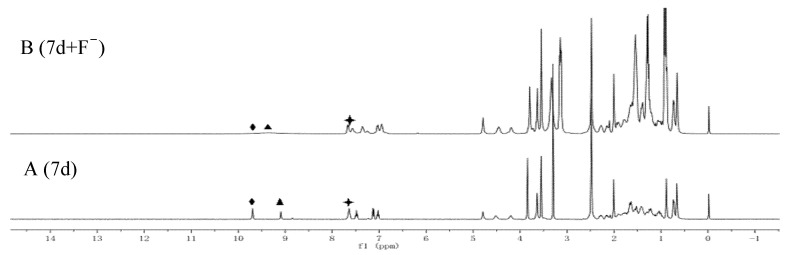
(**A**) ^1^H NMR spectra of chiral molecular tweezer 7d in DMSO-d_6_; (**B**) ^1^H NMR spectra of chiral molecular tweezer 7d affter adding 1 equiv F^−^ ions in DMSO-d_6_. (♦, CH_3_O-PhCONH; ▲, 3α-OCONH; 🟄, 12α-OCONH+ ArH).

It is difficult to accurately describe the recognition mechanism of the chiral molecular tweezer 7d for F^−^ ions only through UV titration and naked-eye recognition test. In general, the complex mechanism of chiral molecular tweezer 7d with anions can be explained more intuitively by observing changes in proton signals and chemical shifts in ^1^H NMR spectra [38,39]. Therefore, in order to further ascertain the mechanism of interaction between the chiral molecular tweezer 7d and F^−^ ions, changes in ^1^H NMR spectra in deuterium dimethyl sulfoxide before and after the addition of F^−^ ions were observed. As shown in Figure 5, the chiral molecular tweezer 7d without F^−^ ions shows two clear signal peaks at 9.70 and 9.09 ppm, which correspond to the amide group N-H linked to 3α-sites of the benzene ring and methyl deoxycholate. In addition, the sharp triplet peak at 7.64 ppm corresponds to the amide group N-H on 12α-sites of methyl deoxycholate and C-H group of the benzene ring. When 1.0 equiv. of F^−^ ions was added to the chiral molecular tweezer 7d, the peak of N-H amide group linked to the benzene ring disappears, and the proton peak of N-H amide group linked to the 3α-sites of methyl deoxycholate shifts up field to 9.33 ppm (Δδ = 0.24), accompanied with significant broadening and weakening of the signal peak. Moreover, the shape of the peak at 7.64 ppm changes significantly, which decomposed from the original triple peak to a dual peak at 7.67 ppm and a single peak at 7.57 ppm. This may be due to the interaction between the active hydrogen in the host molecular tweezer 7d and highly electronegative F^−^ ions, which form hydrogen bonds (N-H... F). As a result, the charge of the benzene rings increases, indirectly leading to the change of aromatic ring hydrogen signal [40]. Therefore, both N-H amide groups on the chiral molecular tweezer 7d interact with F^−^ ions, promoting changes in the optical properties, thus achieving F^−^ ion detection.

### 2.6. Theoretical Calculation

**Figure 6:**
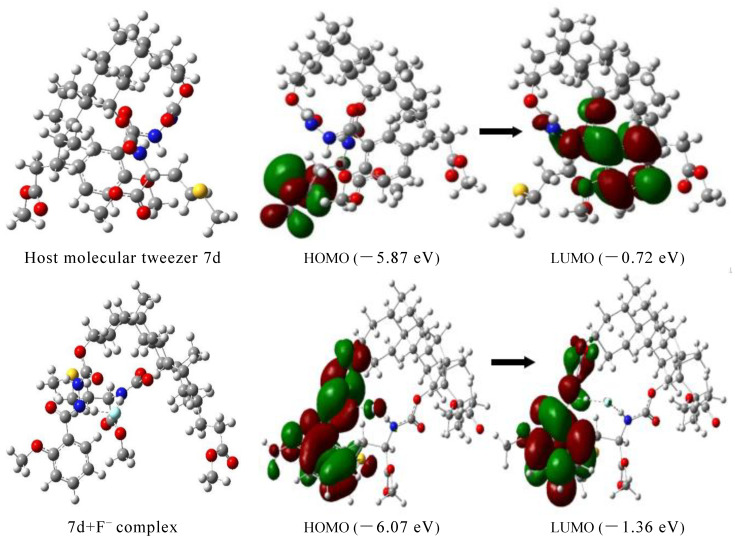
Optimal structures of the host molecular tweezer 7d and 7d + F^−^ complex and the corresponding energy difference of the frontier molecular orbital HOMO-LUMO.

**Figure 7:**
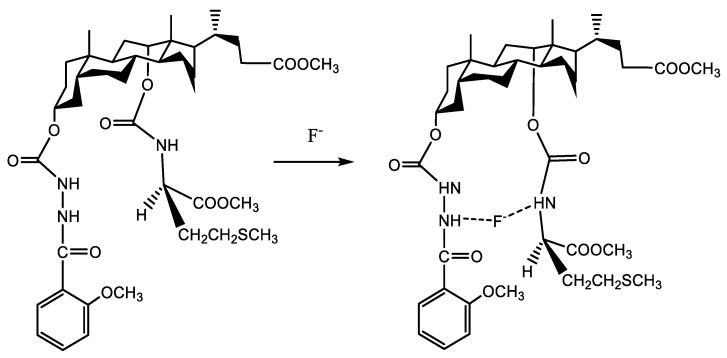
Probable biding mode between the host molecular tweezer 7d and F^−^ ions.

In order to further elaborate the binding mechanism of the host molecular tweezer 7d and anions, its structure before and after the addition of F^−^ ions was optimized using DFT/B3LYP/6-311G method in the Gaussian 09 quantum chemistry software. Figure 6 shows HOMO and LUMO orbital energy and energy level difference [41]. Analysis of the energy-minimized structure of the compound forms by the combination of the host molecular tweezer 7d and F^−^ ions, the energy-minimized conformation of the host molecular tweezer 7d is a tweezer shape, which can provide enough space for the embedment of F^−^ ions. When F^−^ ions interact with the host molecular tweezer 7d, the conformation of the aromatic rings on the host arm changes. Thus the guest F^−^ ions can enter the cracked cavities of the molecular tweezer 7d more conveniently and form a stable complex. F^−^ ions usually have a high electron density and are easy to attract hydrogen atoms. In the presence of the electron-attracting amide group N-H, F^−^ ions act as good hydrogen bond donors. The electron density of LUMO orbital of the host molecular tweezer 7d is mainly distributed on the benzene ring groups, while the electron density of HOMO orbital is mainly distributed on the -SCH_3_ groups. After the addition of F^−^ ions, the electron density on LUMO orbital of the host molecular tweezer 7d and F^−^ complex is mainly distributed on the benzene ring structure units; in contrast, the electron density on HOMO orbital is mainly distributed on the benzene rings and amide N-H groups, and slightly distributed on the methoxy groups linked to the benzene rings. This electron cloud distribution is advantageous to the transfer of intramolecular charge between the aromatic rings and hydrogen bonds, thus leading to changes of UV–vis absorption spectra. According to theoretical calculations, the length of the N-H bond in the host molecular tweezer 7d before the addition of F^−^ ions is 1.016 Å; after the addition of F^−^ ions, the bond length increases (1.042 Å), the distance of H…F is 1.555 Å, and the included angle of N—H…F is 174.3°, indicating that strong intermolecular hydrogen bonds occur between F^−^ ions and the host molecular tweezer 7d (Figure 7). This result is consistent with the change of active hydrogen in the nuclear magnetic titration test. In addition, HOMO-LUMO orbital energy level difference between the host molecular tweezer 7d and 7d + F^−^ complex decreases from 5.14 to 4.71 eV, indicating that the interaction between the host molecular tweezer 7d and F^−^ ions effectively improves the stability of the complex.

### 2.7. Quantitative Determination of F^−^ in Actual Samples

Based on the above mechanism investigation, the chiral molecular tweezer compound 7d has a good response to F^−^ ions. In order to verify the detection ability of the chiral molecular tweezer 7d to F^−^ ions in actual samples, five different food samples were randomly selected and their F^−^ contents were determined according to the standard curve (Table 3).

As listed Table 3, F^−^ ion contents in *Ribes stenocarpum* Maxim, quinoa, Chinese wolfberry, milk powder, and wheat flour are 2.44, 4.68, 2.57, 2.23, and 1.77 mg/kg, respectively. In addition, based on the above results, two kinds of food samples were randomly selected to test the recovery rate of F^−^ ion content by the standard addition method. After the separate addition of F^−^ standard solution of varying F^−^ ion concentrations (2 and 5 mg/kg) to the food samples, UV–vis absorbance values were measured. The results are listed in Table 4, where the recovery rate is in the range of 97–106%, and the relative standard deviation is in the range of 0.38–4.59%. Compared with F^−^ ion selective electrode method in the Chinese national standard GB/T 5009.18, i.e., “Determination of Fluorine in Food”, the relative error between our test results is less than 5% (*n* = 3). Hence, our proposed method can accurately and quantitatively (16–192 μmol/L) detect F^−^ ions in food samples, and has high potential value in real life.

## 3. Materials and Methods

### 3.1. Chemical Reagents and Instruments

All chemicals were purchased from Sigma and Aladdin reagent company without further purification except stated otherwise. All the organic solvents were of analytical grade. The anionic solution is prepared from their various tetrabutylammonium (TBA) salts. Different food samples, namely, milk powder, wolfberry, quinoa, and wheat flour, were bought from the local market (Xining, China), The *Ribes stenocarpum* Maxim were picked from Menyuan, Qinghai province.

Chemical reactions were conducted by MCR-3 microwave chemical reactor (Zheng-zhou, China); Ultraviolet spectra were measured by UV-2600 Shimadzu ultraviolet–visible (Kyoto, Japan); The ^1^H-NMR spectra were recorded with the AVANCE NEO nuclear magnetic resonance spectrometer (Rheinstetten, Germany). The microscopic images were observed with the JSM-7900F field-emission scanning electron microscope (Kyoto, Japan).

### 3.2. Preparation of Cholesteric Molecular Tweezer 7d

Methyl deoxycholate [42] was prepared from deoxycholic acid by methyl esterification in the presence of concentrated sulfuric acid as a catalyst, and methyl l-methionine hydrochloride [43] was prepared by heating and reflux reaction with methanol and SOCl_2_ in the presence of L-methionine. Finally, the cholesteric chiral molecular tweezers were synthesized by microwave irradiation with methyl deoxycholate as a spacer, and arylhydrazine and amino acid as arms. The mass, hydrogen, and carbon spectra of the compound are consistent with those reported in reference [44]. The synthesis route of the molecular tweezer 7a is shown in Scheme S1 (Supplementary Materials).

### 3.3. Solution Preparation and Spectral Analysis

DMSO solutions containing host molecular tweezer 7d (2 × 10^−4^ mol·L^−1^) and different anions of tetrabutylammonium salt (F^−^, Cl^−^, Br^−^, I^−^, HSO_4_^−^, NO_3_^−^, H_2_PO_4_^−^, or Ac^−^) (0.01 mol·L^−1^) were prepared separately at room temperature [45]. A certain amount of DMSO solution containing one type of anion was added to the prepared molecular tweezer solution at room temperature. For the obtained eight mixed solutions, the UV–vis absorption spectra and fluorescence spectra were determined to investigate the selectivity, anti-interference, UV titration results, and Job’s plots of molecular tweezer 7d.

Fluorescence was detected in the range of 200~600 nm. The sample cell for the fluorescence spectrum determination was a quartz cuvette with dimensions of 1 cm × 1 cm × 4 cm. The excitation slit width was 5.0 nm, the emission slit width was 5.0 nm, the excitation wavelength was 270 nm, and the sensitivity was 1. Changes in UV–vis absorption spectra were detected in the range of 200–400 nm using a UV spectrophotometer with a quartz cuvette of 1 cm × 1 cm × 4 cm.

### 3.4. Selective Recognition of Anions by the Host Molecular Tweezer 7d

In a typical run, 0.5 mL DMSO solution containing a certain anion of tetrabutylammonium salt (F^−^, Cl^−^, Br^−^, I^−^, HSO_4_^−^, NO_3_^−^, H_2_PO_4_^−^, or Ac^−^) was added to 0.5 mL of the host molecular tweezer 7d solution of 2 × 10^−4^ mol·L^−1^ (dissolved in DMSO). The mixed solution was corrected to a constant volume of 5 mL. The solution colors, fluorescence emission spectra, and UV–vis absorption spectra for different anions were observed.

### 3.5. Interference Ability of Coexisting Ions and Response Time for Specific Recognition of F^−^

The DMSO solutions containing F^−^ ions and other anions with a molar quantity of 50 times that of the host molecular tweezer 7d were successively added into the host molecular tweezer 7d solution (20 μmol·L^−1^), and the UV–vis absorption spectrum of the mixed solution at 288 nm were measured.

To determine the fluorescence response time of compound 7d to F^−^ ions, 0.5 mL DMSO solution containing 2 × 10^−4^ mol/L the molecular tweezer 7d was added to 0.5 mL F^−^ ionic solution, and the total volume reached 5 mL by adding DMSO, then the fluorescence intensity at 273 nm was measured every 20 seconds until the fluorescence was stable. The slit width of excitation and emission were both 5 nm.

### 3.6. UV—Vis Spectral Titration and Job’s Plot Test for the Recognition of F^−^ by the Host Molecular Tweezer 7d

The DMSO solution containing F^−^ ions was added dropwise to the host molecular tweezer 7d solution of 5.0 × 10^−4^ mol/L, and the UV–vis absorption spectra of the complex solution with different F^−^ ion concentrations (16–192 μmol/L) were determined. The association constant of the host molecular tweezer 7d with F^−^ ions was calculated based on the absorbance values of the complex solutions at 288 nm. Moreover, the standard curve equation was obtained by plotting the graph of F^−^ ions concentration versus the UV–vis absorbance value during the formation of complex. Then, according to the formula LOD = 3 σ/ K, the detection limit of F^−^ ions by the host molecular tweezer 7d could be calculated. Here, in the formula LOD = 3 σ/k, σ is the standard deviation, and k is the slope of linear fitting.

The total concentration of the host and guest F^−^ was kept at 1 × 10^−5^ mol/L, and a series of mixed solutions with different molar ratios (host molecular tweezer 7d to F^−^) of 1:9, 2:8, 3:7, 4:6, 5:5, 6:4, 7:3, 8:2, and 9:1 were prepared successively. The absorbance values of these mixed solutions were measured to obtain the Job point diagram of the complex, and thus the complexing ratio of the complex was determined.

### 3.7. Microstructure Observation and Energy Spectrum Imaging Analysis of the Complex

In order to observe the interaction between the host molecular tweezer 7d and F^−^ ions more intuitively, a field emission scanning electron microscope (FESEM) was used to conduct microstructure observation and energy spectrum imaging analysis on the host molecular tweezer 7d and its complexes with F^−^ ions.

### 3.8. Recognition Mechanism of F^−^ Ions by the Host Molecular Tweezer 7d

According to the composition ratio of the complex, the host molecular tweezer 7d and F^−^ ions in DMSO-d_6_ were quantitatively added to obtain the mixed solution. Changes in ^1^H spectra after the addition of F^−^ ions into the host molecular tweezer 7d solution were observed, and the combining sites of the host molecular tweezer 7d and F^−^ ions and the possible driving force for recognition were determined according to the chemical shift changes of protons on different functional groups. Gaussian 09 software was used for density functional theory (DFT) optimization at B3LYP/6-311G(d) level, as well as simulating the structure of the host molecular tweezer 7d and the molecular structure of the host molecular tweezer 7d and F^−^ ions.

### 3.9. Detection of F^−^ Ions in Actual Food

Total of 4 g *Ribes stenocarpum* Maxim, 4 g quinoa, 4 g milk powder, 4 g flour, and 4 g Chinese wolfberry were weighed respectively and added into a nitrifying tube. Then, 0.2 g copper sulfate, 6 g potassium sulfate, and 10 mL sulfuric acid were added into the nitrifying tube successively. After digestion for 40–50 min at 400 °C, the mixture was centrifuged to obtain the supernatant [46]. Then, 0.5 mL DMSO solution containing the host molecular tweezer 7d (2 × 10^−4^ mol·L^−1^) was added to 0.5 mL supernatant sample solution, and the mixed solution was corrected to a constant volume of 5 mL. The absorbance value of the sample was measured using a UV-spectrophotometer.

## 4. Conclusions

In summary, the chiral molecular tweezer 7d was synthesized via the deoxycholic acid. UV spectrophotometry found that the molecular tweezer 7d had selective recognition ability towards F^−^ ions. The addition of F^−^ ions promoted regular changes of the UV spectrum of the molecular tweezer 7d, and other anions had little interference. The molecular tweezer 7d had a high sensitivity and selectivity towards F^−^ ions. The association constant was 1.72 × 10^3^ L/mol, and the detection limit was 5.14 μmol/L, which was more sensitive than that in many reported methods.

In DMSO solution, addition of F^−^ ions altered the color of the host molecular tweezer 7d solution from colorless to yellow, while the addition of other anions had no effect. Based on this, the rapid naked-eye detection of F^−^ ions was achieved. Furthermore, the complexing process between the host molecular tweezer 7d and F^−^ ions was explored by Job experiment, where the host 7d and F^−^ ions formed a complex in 1:1 ratio.

In order to further clarify the recognition mechanism of the molecular tweezer 7d to F^−^ ions, the NMR spectra and microstructure changes of the molecular tweezer 7d before and after addition of F^−^ ions were studied. It was found that F^−^ ions readily entered the cracked cavities of the molecular tweezer 7d and formed a stable complex. The 12α-CONH and -PhCONH of the host molecular tweezer 7d were the main binding sites, and F^−^ ions were recognized by the intermolecular hydrogen bonding with hydrogen atoms at the amide N-H groups.

The molecular tweezer 7d was applied to the detection of F^−^ ions in actual food samples. Based on the determination of F^−^ ions in quinoa, *Ribes stenocarpum* Maxim, Chinese wolfberry, milk powder, and wheat flour, it was confirmed that a detection method for F^−^ ions in actual food samples in less than an hour was possible. Compared with other reported methods, our method is more rapid and efficient, and the required instruments and equipment are of low cost with simple operation process. It has high selectivity for F^−^ ions, and the recognition results are reliable. Therefore, based on our method, it is expected to develop F^−^ rapid detection kit for its detection in food, environment, and other fields.

## Figures and Tables

**Table 1:** Influences of coexisting ions on the recognition of F^−^ ions by host molecular tweezer 7d.

Coexisting Ion	C_Coexisting Ion_ (mol/L)	(A−A_0_)/A_0_ (%)
Br^−^	8.33 × 10^−4^	−7.14
H_2_PO_4_^−^	8.33 × 10^−4^	−4.29
Cl^−^	8.33 × 10^−4^	2.86
Ac^−^	8.33 × 10^−4^	−2.86
HSO_4_^−^	8.33 × 10^−4^	−7.43
NO_3_^−^	8.33 × 10^−4^	1.43
I^−^	8.33 × 10^−4^	−5.71

**Table 3:** Comparison of F^−^ ion determination results in food samples by our proposed new method and the selective electrode method (*n* = 3).

Samples	New Method (mg/kg)	RSD (%)	Selective Electrode Method (mg/kg)	Relativeerror (%)	*p*
Quinoa	2.45 ± 0.029	1.17	2.41 ± 0.013	1.67	0.83
*Ribes stenocarpum* Maxim	4.68 ± 0.032	0.68	4.50 ± 0.010	4.02	0.94
Chinese wolfberry	2.57 ± 0.042	1.63	2.50 ± 0.006	2.65	0.63
Milk powder	2.23 ± 0.016	0.71	2.18 ± 0.008	2.20	0.88
Wheat flour	1.77 ± 0.028	1.57	1.70 ± 0.006	3.83	0.33

*p* < 0.05, significant difference.

**Table 4:** Determination results of recovery rate of F^−^ ions in food samples after addition of F^−^ standard solution (*n* = 3).

Samples	Add (mg/Kg)	This Method (mg/kg)	Recovery (%)	RSD (%, *n* = 3)	Selective Electrode Method (mg/kg)	Recovery (%)	RSD (%, *n* = 3)	*p*
Quinoa	2	4.66 ± 0.071	105	1.53	4.51 ± 0.009	98	2.29	0.62
	5	7.61 ± 0.073	102	0.95	7.27 ± 0.008	102	3.86	0.60
Chinese wolfberry	2	4.61 ± 0.041	101	0.88	4.77 ± 0.015	106	1.66	0.26
	5	7.57 ± 0.028	100	0.38	7.61 ± 0.030	102	4.59	0.71

*p* < 0.05, significant difference.

## Data Availability

The data presented in this study are available in the article and Supplementary Materials.

## Supplementary Materials

The following supporting information are available online. Scheme S1: The synthetic route of molecular tweezers 7d.

## Sample Availability

Samples of compounds 7d (3α-[o-methoxy-1-(*N*-formyloxyamino)] benzoylhydrazine-12α-[2-(*N*-formyloxyamino)-4-methylthio] methyl butyrate-5β-24-cholic acid methyl ester) are available from the authors.
