# Supplementary material for: Cholesteric Chiral Molecular Tweezer for Rapid Detection of F− in Food Samples
Source: Molecules. 2022 Feb 7;27(3):1098. doi: 10.3390/molecules27031098 (PMC8839199; doi:10.3390/molecules27031098)
Supplement: Supplementary file 1 [file molecules-27-01098-s001.zip › molecules-1572421-supplementary.pdf]

Supporting Information

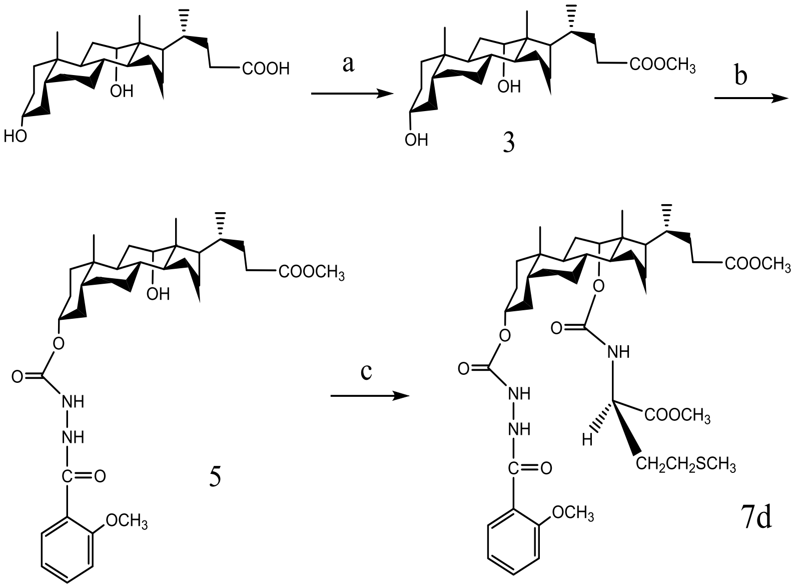

Scheme S1.The synthetic route of molecular tweezers 7d

Reagents and conditions: (a) CH_3OH, H_2SO_4; (b) N_2H_4•H_2O, triphosgene, CH_2Cl_2, MWI; (c) triphosgene, CH_2Cl_2, pyridine, MWI; L-methionine acid methylesters hydrochloride, pyridine, MWI.
